# Retrospective analysis of patients with hereditary nonpolyposis colorectal cancer (HNPCC)

**DOI:** 10.1186/1897-4287-13-S1-A8

**Published:** 2015-09-09

**Authors:** Banaszkiewicz Zbigniew, Pawel Jarmocik, Marcin Mrozowski, Arkadiusz Jawien

**Affiliations:** 1 Department of General, Gastrointestinal, Colorectal, and Oncology Surgery, Department and Clinic of Vascular Surgery and Angiology, University Hospital 2, Bydgoszcz, Poland

## 

We performed a one–center cohort retrospective analysis of 1378 non-selected patients operated for colorectal cancer (CRC) in the years 1994 – 2013. For the purpose of this study we divided patients into three subdivisions reflecting their family history of HNPCC–associated cancers among first- and second-degree relatives. On detailed pedigree analysis of the families we identified 59 patients as being affected with HNPCC (4.28%). Compared with other CRC patients our HNPCC subjects were significantly younger at time of diagnosis (median age 51 years, p < 0.05), were more likely to present with right–sided tumors rather than with rectal ones (p < 0.05). Synchronous tumors were found in 45 CRC patients (3.27%), of whom HNPCC subjects were predominant (8.47 vs. 3.03%, p= 0.02). HNPCC patients were also more likely to receive radical resection surgery compared with other CRC patients (93.22% vs. 86.73%). Patients with HNPCC presented with more favorable staging and more frequent mucinous component on histological examination. Overall survival was statistically longer in HNPCC than in other CRC patients (45 vs. 25 months, p =0.01806). Submitted data and our experience in colorectal surgery made us to support conclusion corroborated by literature evidence and regarding fine distinction between HNPCC and sporadic CRC patients.

